# Engaging Institutional Stakeholders to Develop and Implement Guidelines for Recruiting Participants in Research Studies Using Social Media: Mixed Methods, Multi-Phase Process

**DOI:** 10.2196/23312

**Published:** 2021-10-08

**Authors:** Elizabeth Flood-Grady, Lauren B Solberg, Claire Baralt, Meghan Meyer, Jeff Stevens, Janice L Krieger

**Affiliations:** 1 STEM Translational Communication Center College of Journalism and Communications University of Florida Gainesville, FL United States; 2 Clinical Translational Science Institute University of Florida Gainesville, FL United States; 3 Department of Community Health and Family Medicine University of Florida Gainesville, FL United States; 4 University of Florida Health University of Florida Gainesville, FL United States; 5 University of Florida Health Web Services, Communications & Information Technology University of Florida Gainesville, FL United States

**Keywords:** social media, research recruitment, stakeholder engagement, health communication

## Abstract

**Background:**

Limited regulatory guidance surrounding the use of social media channels for participant recruitment is an interdisciplinary challenge. Establishing stakeholder-informed procedures is essential for ethical and effective use of social media for participant recruitment.

**Objective:**

This study aims to provide replicable procedures for developing and implementing guidelines for using social media to recruit participants in research studies.

**Methods:**

Social media use cases at the university were used to identify institutional stakeholders for the initiative. After establishing workflow procedures, a scoping review of web-based materials about recruitment and research on the internet and social media from 19 peer institutions and 2 federal agencies was conducted to inform the structure of the policies and procedures. End users (investigators and study coordinators; N=14) also provided feedback on the policies and procedures and implementation.

**Results:**

Representatives (n=7) from 5 institutional offices and 15 subject-matter experts from 5 areas were identified as stakeholders in the development of policies and procedures. Peers with web-based materials (n=16) identified in the scoping review revealed 4 themes that served as a basis for developing our policies and procedures. End user feedback further informed the policies and procedures and implementation. A centrally managed social media account for communicating with participants and hosting advertising campaigns on social media was also established and, when combined with the policies and procedures, resulted in 39 advertising campaigns, and 2846 participants were enrolled in health and clinical research studies.

**Conclusions:**

Our policies and procedures allow research teams to harness the potential of social media to increase study recruitment and participation; the transparent, stakeholder-informed process can be replicated by institutional administrators to establish policies and procedures that meet the interests and needs of their research community.

## Introduction

### Background

Social media hold great potential for recruiting prospective participants (ie, identifying, reaching, and advertising studies) into research studies (eg, social and behavioral research, observational studies, and clinical trials) [1]. These channels enable the dissemination of rich information, such as pictures, videos, and links to news articles, which plays an important role in attracting attention and engaging large audiences in dialog about health [2,3]. A recent study of Clinical Translational Science Association web-based strategies for communicating about clinical research participation found that among Clinical Translational Science Associations that hosted separate participant-facing websites, half of those sites linked to social media accounts [4], presumably to direct community members to additional information about research.

Research teams also engage in social media directly for study recruitment. For instance, researchers used Twitter to recruit mothers to participate in a web-based study by tweeting their study invitation and asking users to retweet that information [5]. Teams also used Facebook advertising campaigns to cost-effectively recruit women into human papillomavirus vaccine effectiveness studies [6], screen and recruit adult smokers and heavy-drinking smokers into treatment research [7], and enroll rural adults into health care intervention development studies [8]. Social media are also cost-effective and successful channels for recruiting certain hard-to-reach and underrepresented populations in clinical research [8-10].

Despite the potential of social media as a recruitment tool, there is limited federal guidance for those interested in using social media for research [11], for study recruitment [12], or for institutional review boards (IRBs) charged with reviewing protocols. The federal regulations governing the review and conduct of research with human subjects that went into effect in 2018 [13,14] do not specifically reference the use of social media in research. Federal agency recommendations on the use of social media tools to conduct research remain minimal [11,15]. For instance, in 2013, the Secretary’s Advisory Committee on Human Research Protection published considerations and recommendations on using the internet to conduct human subjects research, and a National Institutes of Health page with guidance regarding social media tools was last updated in 2016 [16,17]. Institutions are thus left to develop policies and procedures that are consistent with federal and state laws on the ethical use of social media in research, and more specifically, the use of social media for research recruitment.

Developing policies and procedures for recruiting research participants via social media that are consistent with applicable law and ethical practice, respect potential participants and their privacy, and are accessible to end users and those enforcing its implementation is a challenge. In an era where cybersecurity, web-based privacy, and terms and conditions of social media are routinely in the news, institutions and their researchers are right to approach this process with caution. Engaging institutional stakeholders involved in research recruitment is a critical step in establishing informed social media recruitment policies and procedures. Stakeholder engagement (SE) relies on multiple methods to identify agreement and disagreement among individuals (stakeholders) affected by decision-making and determine the potential reasons for these differences [18]. Incorporating stakeholder perspectives to develop policies and procedures promotes transparency, increases the quality and trustworthiness of the information, and helps facilitate the implementation of policies and procedures as intended [18-20], ultimately benefiting patients [19]. SE is particularly beneficial when the individuals involved face a similar problem, acknowledge the issue exists, and organize to fix it [21]. In other words, engaging institutional stakeholders involved and affected by social media research and recruitment decision-making should ensure that the process, implementation, and resulting policies and procedures are informed, transparent, and credible.

### Objective

Our goal is to provide replicable procedures for developing and implementing guidelines, which for the purpose of clarity, we refer to as policies and procedures, for using social media to recruit participants into research studies. This process and the resulting policies and procedures can be used by researchers and administrators to develop and implement policies and procedures that should be tailored to the laws, policies, and practices applicable to other institutions.

## Methods

### Overview

During the fall of 2016, the institution identified the need for a more coordinated approach to address privacy, information security, and other questions pertaining to IRB submissions that included increasingly sophisticated use cases for social media. The social media initiative was established in response to this need. The initiative was led by the institution’s Clinical and Translational Science Institute (CTSI). No aspect of this paper represents an official institutional position on social media research and recruitment or the development of policies and procedures. Below, we describe our methods for completing the 6 phases of the initiative.

### Phase 1: Identify Key Stakeholders

SE is the process of engaging individuals, groups, or members of an organization who are (actual) or may be (potential) affected by decision-making or who can influence implementation [18]. Therefore, the initial step was to identify individuals at the institution who were involved in and affected by social media recruitment and research decision-making. Modeled after the CTSI Scientific Advisory Committee at the university, the Directors of Research Services and Strategy and Planning for the institution’s CTSI (author) used protocols requesting the use of social media in recruitment and research that were under review by the IRB as use cases to identify institutional stakeholders. Use case protocols reflected interest in using an application through Facebook to conduct an intervention through the social media site, contracting a third-party vendor to facilitate social media advertisements for study recruitment, and requesting to use social media channels to recruit participants into research studies.

### Phase 2: Establish Workflow Procedures

Next, the Directors of Research Services and Strategy and Planning for the institution’s CTSI convened stakeholders to establish workflow procedures for developing timely social media policies and procedures. Stakeholder concerns, interests, and workflow needs surrounding social media research and recruitment at the institution were discussed and documented. Communication about the policies and procedures workflow that occurred via email (eg, questions and responses) between the initial (phase 1) and follow-up meetings were documented and included as data. Stakeholder input was synthesized and organized into workflow categories.

### Phase 3: Complete Benchmarking and Scoping Review of Existing Peer Guidance

After establishing workflow procedures, a scoping review of existing web-based policies and procedures surrounding social media research and recruitment at our institution and across several peer institutions and federal agencies was completed. Scoping reviews identify available types and sources of evidence and key concepts in a particular research area, determine the extent, range, and nature of existing research activities, summarize and disseminate research findings, and identify existing research gaps across the literature and policies [22]. The goal of this scoping review was to summarize the key elements of existing social media recruitment policies and procedures, map consistencies or inconsistencies and gaps across available policies and procedures, and identify use cases to serve as a baseline for developing policies and procedures at our institution. Benchmarking with peer institutions provided an opportunity to incorporate the perspectives of peer stakeholders with experience and knowledge in this area to shape the direction and scope of future social media endeavors.

As a large, publicly funded, medical research institution, we engaged in a web search to identify materials from 19 peer medical research institutions and 2 federal agencies (N=21). Guidelines, recommendations, including templates and best practices, and policies identified on the 21 websites and corresponding landing pages that addressed the *internet* or *social media* or *social networking sites* as *venues* or *channels* or *platforms* for conducting research or participant recruitment in their scope were considered relevant and included as data. Thematic analysis techniques were used to identify, organize, and refine themes from the data [23].

### Phase 4: Develop, Review, and Refine Social Media Recruitment Policies and Procedures

The themes identified in the scoping review were used to draft the policies and procedures. Policies and procedures have also been developed in alignment with existing institutional social media policies. The proposed policies and procedures underwent a rigorous yearlong review and refinement by stakeholders identified in phase 1 received feedback from potential users.

### Phase 5: Seek Feedback from End Users

This phase was modeled after the focus or working group method, wherein end users provided feedback on the proposed policies and procedures [22]. The intended audience and primary end users of the policies and procedures were investigators (n=6) and study coordinators (n=8) at the institution who participated in separate, small group discussions where they read a comprehensive draft of the policies and procedures and provided feedback on the content, structure, and implementation. Predetermined questions were used to encourage open-ended discussions about the policies and procedures and to obtain information about stakeholder preferences and opinions in a relaxed, nonthreatening environment [22].

### Phase 6: Policies and Procedures Approval and Implementation

In December 2017, the proposed policies and procedures were submitted to senior university administration for potential endorsement and guidance on which institutional officials would need to approve the policies and procedures before implementation. A process for implementing and evaluating policies and procedures at the university was also included in the proposal.

## Results

### Overview

Early in the initiative, it became clear that the most common social media use cases encountered by the IRB were requests to use social media channels for research recruitment. *Therefore, the focus of the initiative was refined to develop policies and procedures to use social media to recruit research participants.* The results of the six phases of the initiative are presented below.

### Phase 1: Identify Key Stakeholders

Using social media to recruit research participants poses a number of ethical, legal, and practical implications for an institution. Seven representatives from 5 institutional offices were identified as stakeholders. In total, 15 subject-matter experts—those with established credibility and expertise in areas salient to the policies and procedures [22,24] were also identified to participate in the initiative. Stakeholders were ultimately divided into two groups: the Social Media in Research Committee and the Health Communication Social Media in Research Task Force. Table 1 includes office or title, institutional representatives, and subject-matter experts who participated in the initiative and a rationale for their inclusion. Stakeholders are further labeled by their participation as either committee or task force group members. The Director of Strategy and Planning and Director of Research Services for the CTSI co-led the initiative, participated as members of the task force, and were included as subject-matter experts (n=15).

**Table 1:** Stakeholders who participated in the initiative.

Office or title			Group		Rationale for inclusion
**IRB^a^**					
	Chair of Health Sciences, Medical Research Chair and Vice Chair of Social, Behavioral, Educational Research	Committee		By federal regulation, federal guidance, and institutional policy, the IRB reviews recruitment materials for accuracy and to ensure the content presented is not coercive [25].	
**Privacy office**					
	Director of Privacy	Committee		Representatives who oversee privacy understand the dynamic, technical aspects of social media platforms and applicable laws (eg, Health Insurance Portability and Accountability Act) pertaining to the privacy and security of an individual’s information, whose data might be viewed or accessed in the process of advertising via social media.	
	Information Security Office: Information Security Manager	Committee		Those who oversee security and information technology also understand the dynamic, technical aspects of social media platforms and applicable laws (eg, Health Insurance Portability and Accountability Act) pertaining to the privacy and security of an individual’s information, whose data might be viewed or accessed in the process of advertising via social media.	
	General Counsel’s Office: Senior University Counsel for Health Affairs	Committee		Representatives from general counsel understand the dynamics surrounding compliance and conduct with the site’s terms of use or potential infringements on social media users’ rights to free speech by removing comments on posted advertisements. Concerns about compliance and conduct represented intersecting stakeholder interests across general counsel, privacy, and security.	
**Office of Research**					
	Director of Research Operations and Services	Committee		Representatives from research administration leadership ensured that the scope and resulting policies and procedures were generally consistent with the university’s mission and goals.	
**Communications professionals**					
	Associate Director of Communications Communication Specialist; Assistant Manager for Web Services	Task force		Professionals in marketing and strategic communications, and those with social media expertise ensured policies and procedures dovetailed with existing guidance (eg, approval and governance of communications channels) and best practices for external communications representing the institution (eg, adherence to institutional brand standards). Communications professionals also anticipated the technical and practical how-to aspects of implementation, and advised on unique considerations, such as how audiences historically interacted with institutional social media.	
Health Communication scientists			Task force		Research scientists with expertise in communication and health and science translation ensured the policies and procedures were accessible, understandable, and usable by intended audiences.
Bioethics and legal experts			Task force		Experts in medical professionalism and clinical and research ethics ensured policies and procedures addressed the ethical and regulatory considerations when using social media in research.
Recruitment and community engagement specialists			Task force		Individuals directly involved in study development and day-to-day recruitment activities informed the clarity and implementation of the policies and procedures.
Regulatory navigators			Task force		Individuals directly involved in study development and day-to-day recruitment activities informed the clarity and implementation of the policies and procedures.
Investigators and research coordinator end users			Task force		Individuals directly involved in study development and day-to-day recruitment activities informed the clarity and implementation of the policies and procedures.

^a^IRB: institutional review board.

### Phase 2: Establish Workflow Procedures

Committee and task force members agreed on the importance of establishing a central process for developing timely policies and procedures. Stakeholders met and established workflow procedures, which were organized into four categories of objectives: (1) establish a project plan, (2) define the scope of planned policies and procedures, (3) address privacy concerns, and (4) contribute to research. Table 2 includes descriptions of the four workflow categories and strategies identified to achieve the objectives.

**Table 2:** Objectives, strategies, and descriptions of workflow procedures.

Objective	Description	Strategies identified for achieving objectives
Establish a project plan	Identify relevant tasks associated with developing and implementing policies and procedures and assign responsibilities to stakeholders	Identify information needed to make decisions on social media protocols pending approval (IRBa) Establish target timeline for developing the policies and procedures (Office of Research) Complete peer benchmarking to identify available types and sources of web-based policies and procedures from peer research institutions (task force) Identify Taskforce personnel responsible for drafting policies and procedures and establish a coordinated process for receiving committee feedback were also identified as relevant tasks Identify the person or office responsible for approving and implementing social media policies and procedures at the institution (committee) Develop social media recruitment templates for submitting IRB protocols and developing theoretically derived advertisements [8], and a risk matrix with examples of high and low-risk social media recruitment activities and the level of review required for each social media recruitment activity (task force and committee) Establish a cost structure for services (task force)
Define scope of planned policies and procedures	Identify subject matter included in the policies and procedures and policy and procedure classification	Determine if policies and procedures will address social media as a channel for advertising studies and recruiting prospective participants exclusively, or if it will also include social media as a channel for hosting studies, interventions, or data collection Decide if policies and procedures will serve as a formal policy, process, or best practice recommendations
Address privacy concerns	Ensure policies and procedures respect prospective participants’ rights to privacy and respond to potential issues of privacy and security for the institution (eg, the university, health system, and institutional researchers)	Address social media users’ reasonable expectations of privacy Address potential concerns about the collection of sensitive and private information on the internet (eg, what is considered Protected Health Information and de facto private and what is private on the internet) Identify steps for responding and managing the exchange of potentially sensitive information on social media Assess institutional risks and liabilities that may result from developing and implementing social media recruitment policies and procedures Identify any legal or ethical obligations to explain what happens when users click on social media recruitment links in advertisements Identify how to address the changing terms of use and privacy policies on social media sites and ensure teams comply with applicable policies
Contribute to research	Enhance public participation in research and disseminate policy and procedure recommendations and resulting research findings	Increase interest and understanding about research participation and generate recruitment leads to increase participation in institutional studies Identify and track metrics for evaluating social media efficacy across individual and multisite studies Develop evidence-based practices relating to recruitment through social media and publishing academic manuscripts on the policies and procedures process and campaign results

^a^IRB: institutional review board.

### Phase 3: Complete Benchmarking and Scoping Review of Existing Peer Guidance

Members of the Taskforce completed peer benchmarking through a scoping review. At the time the scoping review was conducted, most institutions (16/21, 76.2%) had policies or guidelines web-based surrounding the use of the internet and social media to recruit research participants or to conduct research and collect research data. Four themes emerged across existing peer policies and guidelines regarding recruitment and research on the internet and social media, as identified inductively by the task force. These themes address (1) compliance with platform terms of use; (2) social media as a tool for participant recruitment; (3) participant privacy, confidentiality, and data security; and (4) social media as a venue for research. Textbox 1 presents themes and descriptions identified in the scoping review.

**Themes and descriptions identified in the web-based scoping review of peer policies and procedures.**

**Theme name and description**

- Compliance with platform terms of use: focused on the importance of complying with existing site terms of agreement, terms of use, university policies, and adhering to applicable laws when engaging social media for recruitment and research. Understanding and complying with site terms were described as a joint responsibility between investigators and institutional review boards (IRBs).
- Social media as tool for participant recruitment: described recruitment as direct and indirect communication with prospective participants to advertise a study, including posts or paid study advertisements and 2-way communication via researcher-initiated social media accounts to potential research participants through direct messages. Few policies and guidance included steps associated with purchasing and placing study recruitment advertisements. Developing and reviewing recruitment materials was positioned as joint responsibility of investigators and IRBs.
- Participant privacy, confidentiality, and data security: addressed the need to explain privacy and data security processes (eg, how the data are transmitted and maintained on the web), to address the potential risks to using social media, and to acknowledge the limits to confidentiality while emphasizing steps in place to uphold it. Privacy, confidentiality, and data security were positioned, primarily, as responsibilities of investigators or research teams. Teams were also tasked with explaining this information to the IRB in their protocols and to potential research participants (if interested).
- Social media as venue for research: described social media as a channel for hosting research (ie, the location where the observation or intervention will take place), including directly initiating contact with participants and in some cases, studying information about participants that is available without direct contact.

### Phase 4: Develop, Review, and Refine Social Media Recruitment Policies and Procedures

Members of the task force drafted the policies and procedures based on the stated goals and needs of the committee. The committee provided feedback and input during the drafting process and ultimately determined when the policies and procedures could be presented to senior university administration for review and implementation. The three main themes (sections) in our social media recruitment policies and procedures include (1) compliance with platform terms of use (Table 2); (2) participant privacy, confidentiality, and data security (Table 2); and (3) procedures and considerations for using social media to recruit participants.

Theme 3, procedures and considerations for using social media to recruit participants, described the roles and responsibilities of institutional offices and research teams interested in using social media for recruitment; the permissible types, strategies, and considerations for recruiting participants on social media; and mandatory information to include in the social media management plan. Textbox 2 includes a description of the subcategories included in the procedures and considerations theme. The complete policies and procedures can be found in Multimedia Appendix 1 [14,16,26-30].

**Descriptions of social media recruitment procedures and considerations addressed in policies and procedures.**

**Section 3 subcategories in policies and procedures**

- Review of social media recruitment content: the role of the institutional review board (IRB) in reviewing and approving social media recruitment materials (eg, the IRB will review the content of social media recruitment materials in accordance with existing IRB guidelines for traditional media recruitment, such as flyers and news advertisements) as well as the process for research teams submitting recruitment materials to social media sites for approval and posting study materials on social media was described.
- Permissible types of social media recruiting: this section outlined specific options for interacting with prospective participants on social media for recruitment, including static and interactive recruitment materials, recruiting participants via public and private groups, and considerations for private messaging. The responsibilities of research teams interested in recruiting via public and private groups and two-way communication between research team members and prospective participant on social media (eg, private messaging on Facebook and Twitter direct messages) were also included in this section).
- Hosting social media recruitment campaigns: this section described the social media account options for teams to use to facilitate recruitment advertising campaigns on social media, restrictions surrounding the use of personal accounts to purchase and place initial recruitment materials for studies, and parameters for sharing study recruitment materials posted through official university-approved accounts to personal pages and accounts. Details surrounding use of the official *UF Studies* Facebook page, an official account managed by the CTSI Recruitment Center was also included.
- Screening participants and tracking recruitment: this section addressed the procedures for screening prospective participants recruited through social media (for instance, all screening and data collection must occur off social media, such as via phone call or via a secure, university-approved platform, eg, REDCap [Research Electronic Data Capture]), incorporating a waiver of consent for survey studies, and if applicable, a description of the research team’s plan for storing and using any identifiable data collected.
- Developing a social media management plan: the mandatory information required in the social media management plan as part of the IRB protocol’s recruitment plan and that plans must be approved by the IRB before starting recruitment were described in this section.

### Phase 5: Seek Feedback From End Users

During this phase, end users provided general feedback on the usability of policies and procedures for recruiting prospective participants, and the potential for policies and procedures to inform their current recruitment and research practices. End users provided feedback on the scope, clarity, and implementation of the proposed policies and procedures. End users suggested incorporating a definition of personal health information with regard to recruiting on social media. The Director of Privacy provided the Health Insurance Portability and Accountability Act of 1996 definition of individually identifiable information that was provided to the Health System Communications Office in the past for inclusion in the policies and procedures. To increase clarity, end users asked for a list of university-approved resources as they pertain to recruiting on social media (eg, REDCap [Research Electronic Data Capture] for screening) and for additional information regarding sharing recruitment materials from personal accounts. The need for transparent workflow processes and protocol review procedures for teams submitted to study protocols was discussed as part of the implementation. This feedback, along with suggestions for improving readability (eg, removing acronyms, jargon) were incorporated into the policies and procedures and used to inform its implementation.

### Phase 6: Policies and Procedures Approval and Implementation

#### Policies and Procedures Approval and Implementation Proposed Process

The integrated risk management group at the institution endorsed the policies and procedures and proposed a process for implementation. Policies and procedures were hosted on the Office of Research webpage and the IRB website and the CTSI Recruitment Center’s Research Resources webpage linked to the policies and procedures and related resources. The IRB enforced guidelines with support from the Committee and the CTSI Recruitment Center. The CTSI Recruitment Center activated the workgroup and committee structure to facilitate ancillary review of complex recruitment strategies and other social media use cases that fall outside the guidelines and provide consults and services for investigators regarding social media recruitment. The CTSI Recruitment Center also evaluated the 1-year pilot of the policies and procedures and the *UF Studies* Facebook page to assess the effectiveness and resources required for long-term maintenance.

#### Official UF Studies Facebook Page

The *UF Studies* Facebook page, an official account managed by the CTSI Recruitment Center, was established as channel through which teams were permitted to post recruitment materials. The committee and task force collaborated to develop, secure approval for, and launch the *UF Studies* Facebook page, an official account managed the CTSI Recruitment Center. The page served as a centrally managed resource that was made available for both education and recruitment purposes. Community members and prospective participants were the primary audience, and page objectives included educating the community about research and disseminating information about health and science, including research frequently asked questions, results from institutional studies, opportunities to participate in studies, or join established institution-affiliated research registries.

Researchers interested in using social media for study recruitment were the secondary audience. The policies and procedures provided an option for investigators to work with the CTSI Recruitment Center to advertise studies through this page using a streamlined process or follow the policies and procedures for advertising through other official social media accounts approved by the institution. Social media recruitment templates for submitting IRB protocols, developing theoretically derived advertisements, and tracking recruitment metrics and a risk matrix to determine high- and low-risk social media recruitment activities were developed as part of a toolkit for investigators using this central resource. The full policies and procedures, including a risk matrix, is included in Multimedia Appendix 1. Social media recruitment templates are included in Multimedia Appendix 2 and elsewhere [8].

#### Results of Policies and Procedures and UF Studies Facebook Page Implementation

To date, the CTSI Recruitment Center has managed 39 Facebook advertising campaigns for research teams at the institution. A total of 2846 participants were enrolled in health and clinical research studies as a direct result of implementing the social media recruitment policies and procedures and *UF Studies* Facebook pages.

## Discussion

### Principal Findings and Implications

We developed policies and procedures surrounding the recruitment of research participants on social media because our institutional use cases overwhelmingly reflected requests to use social media as a tool for research recruitment. The SE framework was used to identify and engage representatives from key institutional offices and subject-matter experts to facilitate the development and implementation of our policies and procedures. The results include a replicable process for establishing and implementing policies and procedures, an official channel for research teams to use for study recruitment and communicating with prospective participants about research studies, and enrollment of over 2800 participants into health and clinical research studies.

The SE framework was beneficial for identifying and understanding the needs, interests, and perspectives of diverse stakeholders and influencing multiple interconnected systems at our institution to develop policies and procedures. This theoretical process enabled the realization of similar interests, needs, and concerns among stakeholders. Stakeholders were united by their commitment to protect the rights of participants and to establish and maintain ethical research practices at the university, while being responsive to the needs and interests of research teams and advances in technology. Collaboration among stakeholders to develop key considerations for social media recruitment policies and procedures is an important step in establishing trust.

The central channel for running study recruitment campaigns (ie, placing, hosting, purchasing, and tracking), social media recruiting templates for teams and IRB personnel, and prioritizing research education and dissemination of health and science information to the local community through the central channel reflect the practical outcomes produced through the initiative. The central channel benefits research teams who are interested in using social media for recruitment but who may lack the resources needed to develop and manage study recruitment campaigns effectively. The theoretically driven templates for advertising studies ensure that recruitment messages resonate with intended populations [8], and the investigator templates facilitate the timely submission and review of IRB protocols, benefiting researchers and those charged with reviewing social media protocols. Finally, because institutions use social media to communicate with the public about health topics [31] and social media can generate interest and awareness in research [32], including educating the community about research participation as a top priority could increase research literacy and understanding of the research process, ultimately leading to more informed research participants.

Finally, it is important to remain vigilant to changes in social media terms of use and agreement through a continuous review of the social media channel procedures teams used for study recruitment. Indeed, institutions can and should expect researchers to comply with terms of the platform, IRB, and other regulations, and other applicable laws, policies, or guidelines. Developing and maintaining an established communication structure for responding to complex recruitment strategies, social media use cases that fall outside the guidelines, and the changing terms of use of social sites are important for minimizing risk factors and enforcing ethical research recruitment.

### Limitations and Future Directions

Our policies and procedures address study recruitment on social media, although the bulk of our resources aim to streamline study recruitment on Facebook. We focused on Facebook because it is the largest and most popular social networking site [33], and developing resources for multiple social channels would be labor- and time-intensive. Before developing resources for other social media channels, institutions should identify the diversity of audiences and reach of other channels, and whether a particular site has broad enough reach to merit, devoting that level of resources. Due to the increased number of third-party services (eg, TrialSpark) available for managing participant recruitment across multiple social media sites, institutions would benefit from establishing policies and procedures for using outside vendors for recruitment.

### Conclusions

Our goal was to provide limited parameters and procedures for establishing policies and procedures to recruit participants in research studies using social media. The stakeholder-informed, replicable policies and procedures allow research teams to harness the potential of social media to increase study participation and recruitment, while simultaneously managing the potential risks associated with the ubiquity of these channels. Both theory and practice contributed to the development of policies and procedures, enabling a transparent process for administrators at other institutions to replicate and establish policies and procedures that meets the interests and needs of their research community.

## Appendix

Multimedia Appendix 1 Full policies and procedures with risk matrix.

Multimedia Appendix 2 Institutional review board process template: social media management plan.

## Abbreviations

CTSI: Clinical and Translational Science Institute
IRB: institutional review board
REDCap: Research Electronic Data Capture
SE: stakeholder engagement
