# Supplementary material for: Engaging Institutional Stakeholders to Develop and Implement Guidelines for Recruiting Participants in Research Studies Using Social Media: Mixed Methods, Multi-Phase Process
Source: J Med Internet Res. 2021 Oct 8;23(10):e23312. doi: 10.2196/23312 (PMC8538033; doi:10.2196/23312)
Supplement: Multimedia Appendix 1 [file jmir_v23i10e23312_app1.pdf]

Guidelines for Using Social Media to Recruit Research Participants

Updated: August 24, 2018

Introduction
This document offers general guidelines for research teams planning to use social media to recruit human subjects into research. *Human subject* refers to a living individual about whom an investigator conducting research obtains data through intervention or interaction with the individual or identifiable private information (45 C.F.R. §46.102(f)).[1]

These guidelines have been developed based on existing guidance and other advisory documents (e.g., Secretary's Advisory Committee on Human Research Protections, or SACHRP; Considerations and Recommendations concerning Internet and Human Subjects Research Regulations; peer institution guidelines) and are intended to address foreseeable risks of using social media to recruit human subjects into research studies. The UF IRB's investigator guidelines for the text of recruitment materials also address a number of related issues (see Advertising for Research Subjects). Research teams are encouraged to refer to these documents prior to submitting their protocols and to contact the IRB office if they have specific questions not addressed in these guidelines.

These guidelines are not intended to address the use of social media as a venue for conducting research (e.g., collecting data about or observing online environments, incorporating social media into an intervention, data mining from social media sites); such activities are reviewed on a case-by-case basis by the IRB.

Compliance with Social Media Terms of Use
It is the responsibility of the research team, when designing a protocol, to understand the social media site terms of use, or TOU, university policies and applicable laws and to be aware of any research or recruitment-related restrictions on the social media sites through which they intend to conduct their recruitment activities. This includes a site's advertising, privacy and prohibited content policies. After IRB approval, research teams are responsible for amending their protocols as necessary in accordance with any changes to the site's TOU that affect their recruitment plan, and for confirming their compliance with the site's TOU at each continuing IRB review.

Participant Privacy, Confidentiality & Data Security
Private information is described as information about behavior that occurs in a context in which individuals can reasonably expect no observation or recording is taking place, and information provided by an individual for specific purposes that the individual can reasonably expect will not be made public (45 C.F.R. §46.102(f)).[2] If individuals intentionally post or otherwise provide information on the Internet, such information should be considered public unless existing law and the privacy policies and/or terms of use of the entity/entities receiving or hosting the information indicate that the information should be considered 'private.'"[2]

1. It is the responsibility of the research team, when designing a protocol, to understand the various privacy and data security provisions of social media sites. Research teams must include this information in their social media management plans (see 5.a-i below) and provide this information to potential or enrolled participants as appropriate.

[1] 45 CFR 46. HHS.gov. https://www.hhs.gov/ohrp/regulations-and-policy/regulations/45-cfr-46/index.html. Content last reviewed by OHRP February 16, 2016. Accessed April 13, 2017.
[2] hhs.gov. Attachment B: Considerations and Recommendations concerning Internet Research and Human Subjects Research Regulations, with Revisions. HHS.gov. https://www.hhs.gov/ohrp/sachrp-committee/recommendations/2013-may-20-letter-attachment-b/index.html. Published March 22, 2013. Accessed April 13, 2017

2. In social media or other Internet-based research settings, recruitment information can be forwarded or otherwise accessible to other individuals who may not be part of the intended participant pool. Research teams therefore must exercise caution to appropriately identify the targeted participant population and to ensure the equitable selection of participants.

Procedures and Considerations for Using Social Media to Recruit Participants

1. The IRB will review the content of social media recruitment materials according to existing IRB guidelines for traditional media recruitment such as flyers and news ads.[3] The UF IRB must approve the recruitment materials prior to submission to social media sites for approval and prior to posting any such materials on social media.
2. Permissible types of social media recruiting and related considerations:
    a. **Static recruitment materials** include any post or paid advertisement (e.g., Facebook right-column ads) that do not permit liking, commenting, sharing or other public interactions with potential participants on the social media site. Static recruitment materials that link to an IRB-approved destination do not require a plan for managing interactions on the social media site.
    b. **Interactive recruitment materials** include any post or paid advertisement that permits liking, commenting, sharing or other public interactions with potential participants on the social media site. The plan for managing interactive recruitment materials must be clearly delineated in the social media management plan (see 5.a-i below).
    c. **Recruiting via public and private groups** is permitted. Research teams must be aware of any site restrictions on recruiting participants via groups. If no site restrictions exist, teams should determine if the group has its own rules or requirements regarding research recruitment. If research recruitment appears acceptable, research teams must identify if there is a group moderator and request permission to communicate with and recruit group members. It is acceptable to take this step prior to requesting IRB approval. As part of the IRB approval process, research teams must submit to the IRB the text of the recruitment materials that will be shared with group members and their protocols should note any previously obtained approval from the group moderator, plans to obtain approval from the group moderator, or absence of a group moderator, as well as what the process will be for responding to messages from group members.
    d. **Private messaging** for recruitment, defined as two-way communication between a research team member and a potential research subject using private message features on social media sites (e.g., Facebook messages or messenger, Twitter direct messages), may be considered on a case-by-case basis, and may be subject to a more extensive review. Privacy and other considerations related to such communication must be addressed in the social media management plan submitted to the IRB (see 5.a-i below).
3. Social media recruitment activities must adhere to UF and UF Health social media guidelines[4-5] and must be conducted through an official UF or UF Health social media account.
    a. **Personal social media accounts.** Personal social media accounts cannot be used to purchase or place initial recruitment materials for UF studies. Members of the research team are permitted to share recruitment materials that are posted through official UF-approved accounts to their personal pages and accounts, without changing the post or adding comments.

---

[3] IRB Policies, Guidelines and Guidances » UF IRB » University of Florida. http://irb.ufl.edu/index/irb-policies-guidelines-and-guidances.html. Accessed April 13, 2017.

[4] Social Media Use, Guidelines and Policy Implications » Policies » Human Resource Services » University of Florida. http://hr.ufl.edu/manager-resources/policies-2/social/. Accessed April 21, 2017.

[5] Social Media Guidelines for UF Health » Policies and Procedures » Web Services » University of Florida Health Communications and Information Technology. http://webservices.UFHealth.org/files/2011/07/SocialMediaGuidelines.pdf. Accessed April 21, 2017.

b. **Official UF social media accounts.** Research teams must post recruitment materials through official social media accounts that have been approved by UF and/or UF Health.[6-7] Investigators are responsible for all costs associated with paid advertising campaigns. Two options are available:
    i. **UF Studies Facebook account (streamlined submission/approval).** The CTSI Recruitment Center manages the official "UF Studies" Facebook page as a channel through which investigators can post recruitment materials. The CTSI Recruitment Center will facilitate the posting of IRB-approved ads through the "UF Studies" Facebook page in accordance with these guidelines and UF purchasing procedures, if applicable.
    ii. **Official UF and UF Health social media accounts.** If a research team has its own official UF social media account or has received permission from a social media manager to post recruitment materials through an official UF social media account maintained by a different unit, investigators may instead seek approval to post IRB-approved recruitment materials through those accounts. Investigators should obtain approval from the social media account manager prior to submitting a protocol or revision to the IRB. Decisions on whether to allow research recruitment activities through an official account is at the discretion of the manager overseeing that account. As part of the social media management plan submitted to the IRB, investigators should provide written documentation of approval from the account manager and indicate who will be responsible for managing and monitoring the recruitment activities on that account.

4. Screening prospective participants and tracking recruitment:
    a. No screening of prospective participants can occur directly on social media. All screening and data collection must occur offline (e.g., phone) or via a secure, UF-approved platform (e.g., REDCap, Qualtrics). If recruiting to a behavioral research study (e.g., survey) a waiver of consent should appear on the first page of the IRB-approved survey that the social media post links to.
    b. If any identifiable data will be collected as part of the recruitment process, investigators must describe how they plan to collect, store and use the data.
5. Investigators must submit a social media management plan as part of their protocol's recruitment plan. Plans must receive IRB approval prior to starting recruitment. The social media management plan must include:
    a. A list of all social media sites that will be used for recruiting. The list should include, as applicable:
        i. Official UF or UF Health accounts that will be used for posting recruitment materials or purchasing advertisements, and if needed, documentation of approval from the relevant account manager (see 3.b.ii above).
            1. If the UF Studies Facebook page is the only social media channel that will be used, study teams only need to address items 5a and 5c-e in their social media management plans.
        ii. Public or private groups that will be used for recruitment, and a description of previously obtained approval from group moderators, plans to obtain approval from group moderators, or absence of group moderators.
    b. A statement acknowledging the research team's review of and plans for adhering to the terms of use and advertising, privacy and prohibited content policies of the social media sites to be used for recruitment.
    c. Mock-ups of all planned recruitment materials. This includes static and interactive advertisements, posts, images, banners, tags, pre-determined responses to comments,

---

[6] List of Accounts » UF Social Media. http://socialmedia.ufl.edu/accounts/. Accessed June 6, 2017.
[7] Social Account Directory » UF Health's Social Media Hub. https://social.ufhealth.org/accounts. Accessed June 6, 2017.

messages to groups, and any other advertising elements. Include a clear explanation of the format and placement anticipated for each advertisement. To facilitate real-time campaign optimization, it is recommended that teams submit several variations of social media advertisements (e.g., alternate images or text) for IRB approval.

d. A description of the site where the link associated with the recruitment materials will lead (e.g., where social media users will be directed online when they click on an advertisement), and a screenshot of the landing page.
e. If applicable, targeting criteria to be used for paid advertising campaigns.
f. If applicable, the process for responding to group members' messages (see 2.c above).
g. If applicable, a description of how the research team plans to address privacy, data security and identity verification considerations for private messaging with potential participants on a social media site.
h. If applicable, a description of any identifiable data to be collected through the social media site during the recruitment process, and how such data will be stored and used.
i. A description of which personnel will be charged with posting, monitoring and responding to recruitment-related communication on the social media site, as well as any procedures to monitor the activity of those personnel in accordance with the study protocol as well as UF and UF Health social media guidelines,[4,5] including appropriate monitoring to ensure any posts or comments involving protected health information, or PHI, or pertaining to study participation can be promptly addressed (e.g., study complaints, adverse events). For a definition of PHI and frequently asked questions, refer to the UF Privacy Office website. Personnel should also ensure that ads posted through a Facebook account are only displayed on Facebook (i.e., do not select Instagram, Audience Network or other options when placing the ad).

Review Matrix: Research Recruitment on Social Media

Describes the level of review required for different types of study recruitment activities on social media

Levels of Review	Examples of Study Recruitment Activities
**Simple:** Social media recruitment channels that have been pre-vetted by the UF Privacy, Information Security and General Counsel's offices.	a) Posting recruitment materials through the UF Studies Facebook account.<br>b) Purchasing static (right-column) advertisements through any official UF or UF Health Facebook account (the protocol submitted to IRB for approval must also include a screenshot of an email or other communication indicating that the social media account manager has approved purchasing ads through the account).
**Complex**: Social media recruitment activities that *may* require ad hoc or ancillary reviews as requested by the IRB.	a) Posting *interactive* recruitment materials through any official UF or UF Health Facebook account (the protocol submitted to IRB for approval must include a screenshot of an email or other communication indicating that the social media account manager has approved posting materials through the account).<br>b) Posting recruitment materials to Facebook groups.<br>c) Recruiting via private messaging (i.e., direct messaging to prospective participants for recruiting without the exchange of additional communication) on a social media site (e.g. Facebook messages, Facebook messenger, Twitter direct messages).<br>d) Collection of any identifiable data through a social media site during the recruitment process.<br>e) Use of social media sites other than Facebook for study recruitment.

Note: This matrix does not address the review process for protocols that use social media as a venue for conducting research (e.g., collecting data about or observing online environments, incorporating social media into an intervention, data mining from social media sites); such protocols are considered on a case-by-case basis and require ad hoc or ancillary reviews as determined by the IRB.
