# Supplementary material for: Engaging Institutional Stakeholders to Develop and Implement Guidelines for Recruiting Participants in Research Studies Using Social Media: Mixed Methods, Multi-Phase Process
Source: J Med Internet Res. 2021 Oct 8;23(10):e23312. doi: 10.2196/23312 (PMC8538033; doi:10.2196/23312)
Supplement: Multimedia Appendix 2 [file jmir_v23i10e23312_app2.pdf]

IRB Process Template: Social Media Management Plan

This is Multimedia Appendix 2 to the full manuscript: Flood-Grady E, Solberg LB, Baralt C, Meyer M, Stevens J, Krieger JL Engaging Institutional Stakeholders to Develop and Implement Guidelines for Recruiting Participants in Research Studies using Social Media: A Mixed Methods, Multi-phase Process J Med Internet Res 2021.

Investigator Steps for Advertising via UF Studies Facebook Page

Investigators who want to use the UF Studies Facebook page for recruitment should contact the CTSI Recruitment Center for assistance and include the following information in their IRB protocol's social media management plan for recruitment (see Multimedia Appendix 1 for P&P):

1. List the official UF Studies Facebook page as the site that you will use for recruiting.
2. Include a mock-up of your recruitment materials per Facebook advertising policies and IRB requirements, including all copy and images as well as any variations planned for advertisements to facilitate real-time campaign optimization.
3. Describe where the recruitment materials will link (e.g., study webpage) and provide a screenshot of the landing page.
4. For paid ads and boosted posts, establish an ad budget, schedule, and targeting criteria.
5. If the UF IRB has no concerns, it will approve the recruitment materials for submission.
6. Upon IRB approval, investigators contact the CTSI Recruitment Center to coordinate submission, purchase and placement of recruitment materials through the UF Studies Facebook page.
    a. If Facebook approves and starts running a paid ad without modifications, investigators will submit Facebook's approval notification to the IRB for documentation purposes.
    b. If Facebook disapproves a paid ad, investigators will work with the CTSI Recruitment Center to revise the ad to address any issues described in the disapproval email and resubmit the revised ad to the IRB for approval. Upon the IRB's approval of the revised ad, the revised ad can be resubmitted to Facebook.
